# The SARS-CoV-2 Pandemic Impacts the Management of Swiss Pediatric Intensive Care Units

**DOI:** 10.3389/fped.2022.761815

**Published:** 2022-01-28

**Authors:** Maarja Soomann, Pedro D. Wendel-Garcia, Mark Kaufmann, Serge Grazioli, Marie-Helene Perez, Matthias P. Hilty, Maya C. André, Barbara Brotschi

**Affiliations:** ^1^Department of Pediatric and Neonatal Intensive Care, University Children's Hospital Zurich, Zurich, Switzerland; ^2^Children's Research Center, University Children's Hospital Zurich, Zurich, Switzerland; ^3^University of Zurich, Zurich, Switzerland; ^4^Institute of Intensive Care Medicine, University Hospital Zurich, Zurich, Switzerland; ^5^Department for Anesthesia, Surgical Intensive Care, Prehospital Emergency Medicine and Pain Therapy, University Hospital Basel, Basel, Switzerland; ^6^Division of Neonatal and Pediatric Intensive Care, Department of Pediatrics, University Hospital of Geneva, Geneva, Switzerland; ^7^Pediatric Intensive Care Unit, Lausanne University Hospital, Lausanne, Switzerland; ^8^Division of Respiratory and Critical Care Medicine, University of Basel Children's Hospital, Basel, Switzerland; ^9^Department of Pediatric Hematology and Oncology, University Children's Hospital, Eberhard Karls University, Tuebingen, Germany

**Keywords:** pediatric intensive care unit, children, SARS-CoV-2 pandemic, pediatric multisystem inflammatory syndrome temporally associated with SARS-CoV-2, PIMS-TS, management

## Abstract

The impact of the severe acute respiratory syndrome coronavirus-2 (SARS-CoV-2) pandemic on pediatric intensive care units (PICUs) is difficult to quantify. We conducted an observational study in all eight Swiss PICUs between 02/24/2020 and 06/15/2020 to characterize the logistical and medical aspects of the pandemic and their impact on the management of the Swiss PICUs. The nine patients admitted to Swiss PICUs during the study period suffering from pediatric inflammatory multisystem syndrome temporally associated with SARS-CoV-2 (PIMS-TS) and constituting 14% (9/63) of all SARS-CoV-2 positive hospitalized patients in Swiss children's hospitals caused a higher workload [total Nine Equivalents of nursing Manpower use Score (NEMS) points, *p* = 0.0008] and were classified to higher workload categories (*p* < 0.0001) than regular PICU patients (*n* = 4,881) admitted in 2019. The comparison of the characteristics of the eight Swiss PICUs shows that they were confronted by different organizational issues arising from temporary regulations put in place by the federal council. These general regulations had different consequences for the eight individual PICUs due to the differences between the PICUs. In addition, the temporal relationship of these different regulations influenced the available PICU resources, dependent on the characteristics of the individual PICUs. As pandemic continues, reflecting and learning from experience is essential to reduce workload, optimize bed occupancy and manage resources in each individual PICU. In a small country as Switzerland, with a relatively decentralized health care local differences between PICUs are considerable and should be taken into account when making policy decisions.

## Introduction

Children have been reported to be affected by the severe acute respiratory syndrome coronavirus-2 (SARS-CoV-2) in 2–8% of all reported cases (1–4). In addition, they seem to experience milder disease courses, as only 0.2–2% of all affected children have been severely or critically ill (1, 4–6). In the course of the pandemic clusters of severely affected pediatric patients with symptoms similar, although distinctively different from severe Kawasaki disease were reported in Europe and North America (7–13). This new syndrome was named pediatric inflammatory multisystem syndrome temporally associated with SARS-CoV-2 (PIMS-TS) or multisystem inflammatory syndrome in children (MIS-C) (3, 9). It's clinical and laboratory presentations have been described in depth in a series of case reports and case series by now (7–16). Most of the patients diagnosed with PIMS-TS have been treated according to protocols with corticosteroids or immunoglobulins or both, but a high need for organ supportive measures has been reported (3, 8, 17–20). In addition to having to care for patients with this new syndrome, Swiss PICUs were confronted with organizational issues arising from temporary regulations put in place by the federal council due to the pandemic.

There are eight PICUs in Switzerland, distributed all over the small country of 41,285 square kilometers with 8.6 million inhabitants (21, 22). Two PICUs are located in the French speaking part, six PICUs in the German speaking part and none in the Italian speaking part of Switzerland. The aim of our study is to describe the different logistical and medical challenges facing the Swiss PICUs during the first wave of the SARS-CoV-2 pandemic.

## Materials and Methods

### PICU Burden

All eight Swiss PICUs provided data on organizational characteristics and the situation in their units during the study period. These included general characteristics (total number of beds, total number of admissions, and affiliation to adult hospitals) and characteristics related to the SARS-CoV-2 pandemic (percentage of scheduled admissions, cancellation of interventions, staff recruited to adult wards, and percentage of PIMS-TS patients).

For comparing workloads the Nine Equivalents of nursing Manpower use Score (NEMS) and Riker Sedation-Agitation Scale (SAS) as well as a Swiss derivative of the two, the Swiss Society of Intensive Care Medicine (SSICM) shift categories were used (23–25). These scores are used as standard practice in all PICUs in Switzerland and filled out per patient per shift immediately after every nursing working shift. The routine length of a nursing staff shift is 9 h and planned or unplanned double shifts were not practiced. If unplanned absences occurred, units reduced their bed availability for this shift. NEMS score is well-validated and easier to use than the Therapeutic Intervention Scoring System, therefore the Swiss society of intensive care medicine has established using this scoring system nationwide (26–30). NEMS includes data on interventions as well as of therapies and reflects the workload of nurses and physicians. The score includes the following nine items: basic monitoring, intravenous medication, mechanical ventilator support, supplementary ventilator care, single vasoactive medication, multiple vasoactive medication, dialysis techniques, specific interventions in the intensive care unit (ICU), and specific interventions outside the ICU (23). The higher the NEMS Score, the more nursing manpower was needed during a certain shift or during the whole ICU stay. SAS is used to evaluate a patient's level of sedation and agitation. During each shift each patient is given a score from one to seven ranging from an unarousable patient to a dangerously agitated patient (24). SSICM shift categories integrate NEMS and SAS, category 1A of the SSICM defines the most, and category 3 the least, work-load intensive shift for ICU nurses (25). Details SSICM shift categories are shown in Table 1.

**Table 1:** Classification criteria for the Swiss Society of Intensive Care Medicine (SSICM) shift categories.

**Category**	**1A**	**1B**	**2**	**3**
Criteria	NEMS > 30 or NEMS ≥ 21 and SAS > 5	NEMS 21–30 and SAS ≤ 5 or NEMS 13–20 and SAS >5	NEMS 13–20 and SAS ≤ 5 or NEMS <13 and SAS > 5	NEMS <13 and SAS ≤ 5

*NEMS, Nine Equivalents of nursing Manpower use Score; SAS, Riker Sedation-Agitation Scale*.

### Patient and Population Data

In order to compare the critically ill SARS-CoV-2 pediatric patients to the regular PICU population, data on all patients hospitalized in the eight Swiss PICUs in 2019 was used. The whole year instead of the corresponding time period in 2019 was used to avoid a seasonal bias in the group representing regular PICU patients. This data was drawn from the Minimal Dataset of the Swiss Society of Intensive Care Medicine (MDSi) (31). The Pediatric Index of Mortality 2 (PIM2) was used as an indicator of predicted case severity of the patients with PIMS-TS and the general PICU population (32). Data of the patients with PIMS-TS was retrospectively gathered in all eight Swiss PICUs on patients hospitalized from February 24, 2020, the date on which the first SARS-CoV-2 PCR positive patient was reported in Switzerland, up until the June 15, 2020. Included were all patients with age below 20 years and a positive SARS-CoV-2 polymerase chain reaction (PCR) result or the diagnosis of PIMS-TS according to either the Royal College of Child Health and Pediatrics (33), the World Health Organization (34) or the Centers for Disease Control and Prevention case definitions (35), during the PICU stay. Patient data was collected from chart notes and anonymized in the respective centers with a custom made questionnaire, one center's data was obtained from the Risk Stratification in COVID-19 patients in the ICU (RISC-19-ICU) registry (36). Information was gathered also on the use of anti-inflammatory medications including corticosteroids, intravenous immunoglobulin, anakinra, and tocilizumab, which all have been licensed for use in other conditions but were used off label for PIMS-TS.

General epidemiological data on the SARS-CoV-2 pandemic in Switzerland was drawn from the official website of the Swiss Federal Office of Public Health (FOPH) (37). As no reliable serological data was available at the whole population level, the total number of SARS-CoV-2 PCR positive tests in Switzerland was used in calculations to represent number the SARS-CoV-2 cases despite our PIMS-TS group mostly being PCR negative and seropositive. Data on the demographics of Switzerland was obtained from the website of the Swiss Federal Statistical Office (21).

### Definitions of Organ Dysfunction

Acute respiratory distress syndrome (ARDS) was defined according to the Pediatric Acute Lung Injury Consensus Conference definition for pediatric acute respiratory distress syndrome (38). Shock was defined as an arterial systolic blood pressure below the age adapted 5th percentile or a systolic blood pressure below two standard deviations of the age adapted mean, and/or the need for vasoactive support to maintain blood pressure in the range (39, 40). Myocardial injury was defined as ejection fraction reduced below 55% measured using the biplane Simpson method according to the current pediatric echocardiographic recommendations (41, 42). Renal dysfunction was defined according to the pRIFLE classification (43). Hepatic dysfunction was defined as the elevation of liver enzymes above the age adapted reference and elevation of prothrombin time (44). An abnormal prothrombin time or activated partial thromboplastin time (according to age adapted reference values of the local laboratories) was classified as a coagulation disorder (45).

### Ethical Approval

The study proposal (KEK: 2020-00720), as well as the RISC-19-ICU (KEK: 2020-00322, ClinicalTrials.gov Identifier: NCT04357275) registry have been evaluated by the Cantonal Ethics Committee of Zurich, a member of the Swiss Association of Research Ethics Committees—Swissethics and in line with the Swiss Federal Human Research Act deemed exempt from the need for additional ethics approval. The study complies with the Declaration of Helsinki, the Guidelines on Good Clinical Practice (GCP-Directive) issued by the European Medicines Agency as well as the Swiss law and Swiss regulatory authority requirements and has been designed in accordance with the Strengthening the Reporting of Observational Studies in Epidemiology (STROBE) guidelines for observational studies.

### Statistical Analysis

Statistical analysis was conducted employing the R environment for statistical computing version 4.0.2. Comparisons of population characteristics were performed using Wilcoxon rank-sum test for continuous not normally distributed and the chi-squared test for categorical variables. Continuous not normally distributed data is presented as median with interquartile ranges (IQR) and nominal data as counts and percentages.

## Results

### PICU Burden

The Swiss PICUs varied in general characteristics as well as in characteristics related to the pandemic. The different medical and logistical aspects affecting the PICUs are listed in Table 2. In addition to some PICUs having to care for patients with this new syndrome, all PICUs were confronted with organizational issues arising from temporary regulations put in place by the federal council due to the pandemic. These general regulations had different consequences for the eight PICUs due to their different characteristics. As shown in Table 2, there were units (PICU 8) not affiliated to adult hospitals and without staff recruitment to adult wards, where nevertheless scheduled interventions were canceled due to regulations set by the federal council. As a consequence, this PICU was not fully occupied and had a relatively low workload. Other PICUs (PICU 5), however, were affiliated to an adult ward and staff recruitment to the adult wards was required. Therefore, cancellation of scheduled interventions was essential to have enough manpower and staff for the emergency admissions to these PICUs (PICU 5). Other PICUs (PICU 1) were also influenced by local circumstances with staff being recruited to adult wards due to staff shortage in the area despite the PICU not being affiliated with an adult hospital.

**Table 2:** General characteristics and characteristics related to the pandemic of the eight Swiss pediatric intensive care units between 02-24-2020 and 06-15-2020.

**Characteristics**	**PICU 1**	**PICU 2**	**PICU 3**	**PICU 4**	**PICU 5**	**PICU 6**	**PICU 7**	**PICU 8**
**General characteristics**	
Children hospital associated with adult hospital	No	Yes	Yes	Yes	Yes	Yes	No	No
Number of PICU beds	8	12	9	7	12	11	10	25
ECMO center	No	Yes	No	Yes	Yes	No	No	Yes
Perform hemodiafiltration	No	Yes	No	Yes	Yes	Yes	No	Yes
**Characteristics related to the pandemic**	
Total number of admissions	115	224	99	265	168	156	136	365
Total NEMS points per patient	237	219	262	186	466	164	305	294
Percentage of scheduled admissions	24%	49%	9%	16%	51%	24%	27%	49%
Cancellation of scheduled interventions	Yes	Yes	Yes	Yes	Yes	Yes	Yes	Yes
Staff recruited to adult wards	Yes	No	Yes	Yes	Yes	Yes	No	No
Percentage PIMS-TS patients of all admissions	0.9%	0	0	1.5%	1.8%	0	0	0.3%
Number of positive SARS-CoV-2 PCR patients without PIMS-TS	0	0	0	0	0	0	0	0

*ECMO, extra corporal membrane oxygenation; NEMS, Nine Equivalents of nursing Manpower use Score; PCR, polymerase chain reaction; PICU, pediatric intensive care unit; PIMS-TS, pediatric inflammatory multisystem syndrome temporally associated with severe acute respiratory syndrome coronavirus-2; SARS-CoV-2, severe acute respiratory syndrome coronavirus-2*.

Four out of eight PICUs had patients admitted with PIMS-TS between February 24, 2020 and June 15, 2020. Patients with PIMS-TS constituted 0.6% of all Swiss PICU admissions (9/1,528) during this time period and 0.3–1.8% of the PICU admissions of PICUs treating patients with PIMS-TS. A comparison of patients with PIMS-TS and general pediatric intensive care unit patients hospitalized in 2019 is shown in Table 3, based on quantitative and qualitative measures of case complexity. Patients with PIMS-TS had higher total NEMS scores (*p* = 0.0008) and were classified to higher SSICM workload categories more frequently than general PICU patients hospitalized in 2019 (*p* < 0.0001).

**Table 3:** Comparison of PIMS-TS patients and all PICU patients hospitalized in 2019 based on quantitative and qualitative measures of case complexity.

**Characteristics**	**PIMS-TS patients**	**All patients in 2019**	***p* value**
	***n* (patients) = 9**	***n* (patients) = 4,881**	
	***n* (shifts) = 273**	***n* (shifts) = 63,015**	
**Quantitative measures, median (IQR)**			
Length of stay in days	10 (9-11)	1.6 (0.8–3.9)	<0.0001
Duration of positive pressure ventilation in hours	72 (0–99)	0 (0–24)	0.025
PIM2 on admission	4.3 (1.5–7.2)	1.4 (0.6–3.2)	0.07
Total NEMS points	569 (496–736)	92 (51–239)	0.0008
**Qualitative measures, number of shifts (percentage)**			
**SSICM shift categories**			
1A + 1B	156 (57.1)	23,557 (37.4)	0.0001
2 + 3	117 (42.9)	39,458 (62.6)	
**Riker SAS**			
SAS ≥ 5	33 (12.1)	6,523 (10.4)	0.35
SAS <5	240 (87.9)	56,492 (89.6)	

*IQR, interquartile range; NEMS, Nine Equivalents of nursing Manpower use Score; PICU, pediatric intensive care unit; PIM2, Pediatric Index of Mortality 2; PIMS-TS, pediatric inflammatory multisystem syndrome temporally associated with severe acute respiratory syndrome coronavirus-2; SAS, Sedation-Agitation Scale; SSICM, Swiss Society of Intensive Care Medicine*.

### Epidemiology of SARS CoV-2 Positive Patients

The total of 1,113 children and young adults under the age of 20 were tested positive for SARS-CoV-2 infection between February 24, 2020 and June 15, 2020 in Switzerland, of which 6% (63/1,113) had to be hospitalized [Swiss FOPH (37)]. Ten pediatric patients with a concurrent positive SARS-CoV-2 PCR result or with PIMS-TS were hospitalized in PICUs. The only patient not diagnosed with PIMS-TS was admitted to the PICU due to an acute necrotizing encephalopathy. As the role of SARS-CoV-2 in the pathogenesis and disease progression remained unclear in this case, this patient's data was excluded from the present analysis. The nine remaining patients with PIMS-TS hospitalized in Swiss PICUs constituted 14% (9/63) of all SARS-CoV-2 positive hospitalized and 1% (9/1,113) of all SARS-CoV-2 positive children and young adults under the age of 20 in Switzerland during that period [Swiss FOPH (37)]. The incidence of severe PIMS-TS requiring hospitalization at a PICU was, therefore, 0.5 per 100,000 people under the age of 20 during the study period. No information is available on seropositivity in the general population during the study period. The general characteristics of all the PIMS-TS patients, details on organ dysfunction and applied therapies are presented in Table 4.

**Table 4:** General characteristics of patients with pediatric inflammatory multisystem syndrome temporally associated with severe acute respiratory syndrome coronavirus-2 (PIMS-TS), details on organ dysfunction and applied therapies.

**Characteristic**	***n* (%)**	**Median (IQR)**
**General characteristics**	
Age in years		11 (9–11.5)
**Gender**	
Female	2 (22%)	
Male	7 (78%)	
Body mass index in kg/m^2^		20 (16.8–26.4)
**Organ dysfunction**	
Acute respiratory distress syndrome	2 (22%)	
Shock	8 (89%)	
Myocardial injury	4 (44%)	
Renal dysfunction	6 (67%)	
Hepatic dysfunction	4 (44%)	
Coagulation disturbances	6 (67%)	
**Supportive measures**	
Any type of respiratory support	9 (100%)	
**Highest level of respiratory support**	
Invasive ventilation	5 (56%)	
Non-invasive ventilation (NIV)	1 (11%)	
Continuous positive airway pressure (CPAP)	0 (0%)	
High-flow nasal cannula (HFNC)	1 (11%)	
Low-flow nasal cannula (LFNC)	2 (22%)	
**Duration of respiratory support per patient in days**	
Invasive ventilation (*n* = 5)		3.8 (3–8)
NIV (*n* = 3)		1 (0.4–4)
CPAP (*n* = 0)		0
HFNC (*n* = 2)		3 (2–4)
LFNC (*n* = 3)		1 (1–6.3)
Vasopressors or inotropes	8 (89%)	
Duration in days		3.5 (3-4.8)
Extracorporeal membrane oxygenation (ECMO)	0	
Continuous renal replacement therapy	1 (11%)	
Duration in days		3
Cytokine absorption therapy	1 (11%)	
**Drug therapies**	
Hydroxychloroquine	3 (33%)	
Intravenous immunoglobulin (IVIG)	6 (67%)	
Steroids	6 (67%)	
Biological agents	6 (67%)	
Anakinra	6 (67%)	
Tocilizumab	2 (22%)	
**Combinations of immunotherapies**	
None	1 (11%)	
IVIG only	1 (11%)	
Anakinra only	1 (11%)	
Steroids and anakinra	1 (11%)	
Steroids and IVIG	1 (11%)	
IVIG, steroids, and anakinra	2 (22%)	
IVIG, steroids, anakinra, and tocilizumab	2 (22%)	
**Outcome**	
Patients alive on discharge	9 (100%)	

*IVIG, intravenous immunoglobulins*.

## Discussion

In this observational study, we describe different aspects of the first wave of the SARS-CoV-2 pandemic impacting the management of Swiss PICUs. Many of these aspects are difficult to measure and especially their temporal relationship complicates the analysis. Although, initially the disease was thought to affect predominantly adult departments, in the course of the pandemic, pediatric units and PICUs were more and more affected. Most of the staff were confronted with a pandemic, a new and unknown situation, for the first time in their life.

The absolute number of pediatric patients requiring intensive care due to severe SARS-CoV-2 and PIMS-TS during the study period was low. Similar results of hospitalized children and adolescents admitted to PICUs have been reported in other studies as well (46, 47). Although Switzerland is a small country, eight independent PICUs treat critically ill children. Each PICU is quite small, operating a relatively small number of beds. Although cooperation between the PICUs exists in several forms, every day professional exchange is not common. These circumstances explain why it took time for the PICU staff to learn about the new disease. However, in the course of the first wave of the pandemic, the PICUs recognized, that it is important to cooperate and to exchange experiences with each other to learn fast about the new disease and to improve patient outcome. As a consequence, Swiss consensus guidelines to treat pediatric patients with PIMS-TS for best practice were established by a multidisciplinary group of Swiss pediatric clinicians with expertise in intensive care, immunology, rheumatology, infectious diseases, and hematology during the second wave of the pandemic at the end of 2020 (http://transfer.imk.ch/f.php?h=3R2LIfFV&d=1).

Apart from the challenge of treating this unknown severely ill patient group, there were different logistical and organizational issues influencing the management of the PICUs. At the beginning of the pandemic a variety of measures to prepare the Swiss hospital network for the pandemic were instituted on federal level. Nationwide, scheduled interventions were canceled to reduce the need for post-interventional intensive care and to increase resources for emergency admissions. The federal government did not differentiate between adult and children's hospitals, provided services or bed occupancy of the individual PICUs. In some regions staff recruitment to the adult wards was required, a measure adopted by regional governments. Our data illustrates the effects of those measures on PICUs. For example, the comparison of PICU 5 with PICU 8 shows the different impact of the policy measures on the workload of individual PICUs. PICU 5 and 8 had almost the same, relatively high, percentage of scheduled admissions. At the same time PICU 8 was not associated to an adult hospital, lost no staff to adult wards and treated very few patients with PIMS-TS, whereas PICU 5 was associated to an adult hospital, a part of its staff was recruited to adult wards and treated significantly more children with PIMS-TS. These clearly different circumstances led to different workloads, to some extent illustrated by the difference in average total NEMS points per patient during the study period (466 vs. 294, Table 2). Although small in numbers, PIMS-TS patients admitted to PICUs were severely ill, caused a higher workload then average PICU patients (total NEMS points per patient 569 vs. per general PICU patient 92, Table 3)The total impact of these patients on total PICU workload during this period can unfortunately not be quantified based on our data.

Different measures influenced the workload and the bed occupancy of the individual PICUs differently, depending on the presence or absence of the single factors and their temporal relationships. Due to their complexity, the exact quantification and comparison of the impact of all these factors was unfortunately not possible. However, thanks to the observations and experiences of the first wave, we recognize that the individual PICUs should be organized primarily by a regional and not federal level and in accordance with but not the same to the surrounding adult hospitals. A timely analysis of the burden and duties of the individual PICU is essential to reduce the workload of the PICU and simultaneously ensure optimal bed occupancy for economic reasons. During a pandemic timely customization of measures is also essential to adapt the usual available resources to the present requirements. Although it is very important to learn of the experiences of the different countries regarding management of a PICU during a pandemic, there is not much literature. Zeng et al. give insights on the management of a PICU in the SARS-CoV-2 pandemic in southwest China. They focus on measurements ensuring the safety of both patients and medical staff (48). This paper proposes and optimizes a strategic plan for the management of SARS-CoV-2 outbreak in PICU and use risk management and process control to effectively manage the department as well as to protect both the patients and the staff (48).

Our study has some limitations. The retrospective design restricts the range of data available from the study population, this applies for both the historical comparison cohort as well as the epidemiological PIMS-TS data. The logistical aspects are difficult to measure and their temporal relationship has impact on their influence on the management of a single PICU. However, the aim of this study was to describe the different issues of the pandemic influencing the PICUs in Switzerland, a small country organized in a decentralized manner.

In conclusion, the SARS CoV-2 pandemic does not exclusively affect adult ICUs, with PICUs also having to face a variety of eventualities with a plethora of consequences. As pandemic continues, reflecting and learning from experience and cooperate with other PICUs is essential to reduce the workload, optimize the bed occupancy and dispose the resources in each individual PICU. In a small country as Switzerland, the different PICUs should be organized dependent of the local and not federal health care policy due to their different characteristics.

## Data Availability Statement

The original contributions presented in the study are included in the article/supplementary material, further inquiries can be directed to the corresponding author/s.

## Ethics Statement

The studies involving human participants were reviewed and approved by Cantonal Ethics Committee of Zurich, Stampfenbachstrasse 121, 8090 Zurich. Written informed consent to participate in this study was provided by the participants' legal guardian/next of kin.

## Author Contributions

MS and BB made the conception and the design of the study and draft the manuscript. PW-G and MK performed all statistical analyses of the study, interpreted results, and critically revised the manuscript. SG, M-HP, MH, and MA helped with acquisition of data and critical review of the manuscript. All authors have read and approved the final manuscript.

## Conflict of Interest

The authors declare that the research was conducted in the absence of any commercial or financial relationships that could be construed as a potential conflict of interest.

## Publisher's Note

All claims expressed in this article are solely those of the authors and do not necessarily represent those of their affiliated organizations, or those of the publisher, the editors and the reviewers. Any product that may be evaluated in this article, or claim that may be made by its manufacturer, is not guaranteed or endorsed by the publisher.

## Abbreviations

ARDS: acute respiratory distress syndrome
FOPH: Federal Office of Public Health
ICU: intensive care unit
IQR: interquartile range
MDSi: Minimal Dataset of the Swiss Society of Intensive Care Medicine
MIS-C: multisystem inflammatory syndrome in children
NEMS: Nine Equivalents of nursing Manpower use Score
PCR: polymerase chain reaction
PICU: pediatric intensive care unit
PIM2: Pediatric Index of Mortality 2
PIMS-TS: pediatric inflammatory multisystem syndrome temporally associated with severe acute respiratory syndrome coronavirus-2
pRIFLE: pediatric Risk, Injury, Failure, Loss, End Stage Renal Disease
RISC-19-ICU: Risk Stratification in COVID-19 patients in the ICU
SARS-CoV-2: severe acute respiratory syndrome coronavirus-2
SAS: Sedation-Agitation Scale
SSICM: Swiss Society of Intensive Care Medicine.
